# Robust Face Recognition Using the Deep C2D-CNN Model Based on Decision-Level Fusion

**DOI:** 10.3390/s18072080

**Published:** 2018-06-28

**Authors:** Jing Li, Tao Qiu, Chang Wen, Kai Xie, Fang-Qing Wen

**Affiliations:** 1 School of Electronic and Information, Yangtze University, Jingzhou 434023, China; 201501479@yangtzeu.edu.cn (J.L.); 500646@yangtzeu.edu.cn (K.X.); wenfangqing@yangtzeu.edu.cn (F-Q.W.); 2 National Demonstration Center for Experimental Electrical and Electronic Education, Yangtze University, Jingzhou 434023, China; 3 School of Computer Science, Yangtze University, Jingzhou 434023, China; 201603441@yangtzeu.edu.cn

**Keywords:** face recognition, convolutional neural network, color 2-dimensional principal component analysis, decision-level fusion, normalization, layered activation function, probabilistic max-pooling

## Abstract

Given that facial features contain a wide range of identification information and cannot be completely represented by a single feature, the fusion of multiple features is particularly significant for achieving a robust face recognition performance, especially when there is a big difference between the test sets and the training sets. This has been proven in both traditional and deep learning approaches. In this work, we proposed a novel method named C2D-CNN (color 2-dimensional principal component analysis (2DPCA)-convolutional neural network). C2D-CNN combines the features learnt from the original pixels with the image representation learnt by CNN, and then makes decision-level fusion, which can significantly improve the performance of face recognition. Furthermore, a new CNN model is proposed: firstly, we introduce a normalization layer in CNN to speed up the network convergence and shorten the training time. Secondly, the layered activation function is introduced to make the activation function adaptive to the normalized data. Finally, probabilistic max-pooling is applied so that the feature information is preserved to the maximum extent while maintaining feature invariance. Experimental results show that compared with the state-of-the-art method, our method shows better performance and solves low recognition accuracy caused by the difference between test and training datasets.

## 1. Introduction

Biometric recognition technology is a method for identification or verification based on human physiological or behavioral characteristics. Biometric features for authentication include iris, fingerprint, palm print, face, ear type, voiceprint and gait. Face recognition is face-based identification technology, which is a main trend of biometric recognition. Compared with other biometric technologies, face recognition technology has unique advantages. It does not require user cooperation and the acquisition process is non-contact. Meanwhile, it has better concealment. The benefits make it particularly suitable for security monitoring and airport anti-terrorism applications.

Face recognition is generally divided into three basic technical lines [1]. The first type is 2D face recognition based on pictures. 2D face recognition started earlier and has achieved great success. Before 2003, most of the work about face recognition focused on 2D face recognition, which mainly included: (1) recognition algorithms based on geometric features [2,3]; (2) recognition algorithms based on subspace [4,5]; (3) recognition algorithms based on elastic matching [6,7]; (4) recognition algorithms based on neural networks [8,9]. 2D face recognition identified a face with the brightness image, which is inevitably influenced by factors such as illumination and which is also the main bottleneck for the further development of 2D face recognition. 

The second category is 3D face recognition based on 3D geometric information. In recent years, attention increasingly has been placed on 3D face recognition. Liang proposed a new method to handle pose variations in 3D face recognition [10] and Abbad makes a great contribution to the problem of expression variations in 3D face recognition [11]. Apparently, the remarkable advantage of 3D data over 2D data is that it has the original geometric information of the human face. Two-dimensional face images are easily affected by factors such as lighting, posture, and occlusion, which will lead to a decrease in the recognition ability of the face recognition system. However, a 3D face recognition algorithm is hardly affected by these factors. There is more potential in solving face problems with variable illumination and posture. This is because the three-dimensional data can truly reflect the surface geometric features of the human face and will not lose information due to changes in posture. Moreover, if the acquisition process of three-dimensional data is not considered, since 3D data has no brightness information, the three-dimensional data will not be affected by the change of illumination. Owing to these, 3D face recognition is booming. Dagnes reviews a series of 3D face occlusion recognition methods [12]. Facial occlusions are general situations which is hard to address in 2D face recognition, however, it has been preferably solved in 3D face recognition. Vezzetti proposes a novel automatic method for facial landmark localization relying on geometrical properties of 3D facial surface in the presence of occlusions [13]. In other fields, 3D recognition technique can also be applied. Moos proposed a methodology to automatically diagnose and formalize prenatal cleft lip [14]. Similarly, in the literature [15], Moeini proposed a feature extraction to handle the problem of facial appearance changes including facial makeup and plastic surgery in face recognition. Meanwhile, other scholars make distinctive contributions to 3D face recognition [16,17,18].

The third type of face recognition technology, namely the 2D+3D dual-mode fusion technique [19,20], has achieved good performance. The 2D+3D effectively combines the more mature 2D image recognition technology with the three-dimensional shape attributes of the face to obtain a better face recognition performance. In recent years, there has been progress in 3D-2D face recognition [21,22].

Deep learning has grown rapidly on account of the emergence of large-scale face datasets, whose powerful data learning has brought a research upsurge to 2D face recognition. It can effectively solve the problems of traditional machine learning algorithms. Our work focuses on the application of state-of-the-art deep learning technology in face recognition. 

Deep learning [23] is a hot area in machine learning research by building deep neural networks to simulate the mechanism of human brain and then interpreting and analyzing data such as image, voice and text [24,25]. The effectiveness of traditional machine learning depends largely on the performance of handcrafted feature representation. During this process, the role of machine learning approaches is only to optimize learning weights and ultimately to produce optimal learning outcomes [26]. Different from the traditional machine learning methods, deep learning tries to automatically complete the work of data representation and feature extraction [27,28]. The main power of a convolutional neural network (CNN) lies in the deep architecture, which allows for extracting a set of discriminating feature representations at multiple levels of abstraction. In recent years, CNN has been widely applied in face recognition because of its good performance [29,30]. The success of CNN is attributed to its ability to learn rich image features. However, training a deep CNN network relies on learning millions of network parameters and requires plenty of labeled datasets for pre-training [31].

In practice, we do not usually train an entire CNN network from scratch, since it is impossible to collect a dataset with sufficient size to meet the needs of the CNN for each new face recognition task [32]. As an alternative, it is common to pre-train the CNN model on a large dataset and then use the weights of this model as an initialization or as a specific feature extractor for the task of interest, then finally fine tune the network model using the test set [33,34].

However, when the test and training datasets have great differences in illumination, expression, viewpoints or other factors, even the fine-tuned CNN cannot achieve a good recognition performance. The fundamental cause is that fine tuning is still based on the pre-trained CNN model which limits the recognition performance for the current recognition task. Effective fine tuning requires that all layers of the pre-trained model need to be fine-tuned when the variations between the source and target applications are significant. However, the limited fine-tuned data can cause over-fitting when the pre-trained CNN is fine-tuned too much [35]. CNN completes the classification and recognition task mainly through the study of the distribution of data [36,37]. When there is large difference between test and training set, the initial data distribution obtained by the pre-trained CNN network is difficult to change, which makes it difficult to improve the adaptability of the CNN model even if the CNN model is fine-tuned.

Another problem of the training network is the large number of network parameters and the fact that it is time consuming. The reason is that the parameters’ change of any layer will cause the change of the distributions for input data in subsequent layers, which leads to the neural network constantly needing to adapt to the new data distribution. Thus, tuning parameters carefully and training with smaller learning rate are particularly important. Moreover, non-linear saturation of the activation operation makes it more difficult [38,39]. LeCun proposed that data normalization might speed up network convergence [40]. In 2015, batch normalization was proposed to solve the internal covariate shift in [41].

Aiming at the above problems, we propose a method of face recognition using a color 2-dimensional principal component analysis-convolutional neural network (C2D-CNN) to make full use of color information and complementary information for face recognition.

For color information, there have been some scholars [42,43,44] who have noticed its importance to face recognition. Color face recognition is usually conducted on each color channel and the final result comes from the fusion of the three color channels. Similarly, CNN networks make full use of three color channels of RGB for feature extraction, respectively. All of them omit the inner correlations among color channels. A color channel fusion method using a joint dimensionality reduction algorithm was proposed by [45] which can effectively improve the performance of color face recognition. In contrast, color 2-dimensional principal component analysis (2DPCA) is based on the concept of color value including three channels, which devotes to making full use of color information and the correlation between color channels. Color 2DPCA is devised to combine color and spatial information. It still has good robustness for the test and training set with large differences. So, color 2DPCA provides an effective way to improve the robustness of CNN.

The contributions of this paper are summarized as follows:

- We propose a deep face recognition method, C2D-CNN, which combines the two features into the decision-making level, with high-accuracy and low computational cost.
- We investigate a new CNN model. Through careful design, (1) normalization is introduced to accelerate the network convergence and shorten the network pre-training time; (2) a layered activation algorithm is added to improve the non-linear function of the activation function and solve the problem of gradient saturation and gradient diffusion; (3) probabilistic max-pooling is applied to preserve the feature in maximum extent while maintaining feature invariance.

The remainder of this paper is organized as follows: Section 2 presents the C2D-CNN model; Section 3, the experimental results are analyzed and discussed; the conclusion is given in Section 4.

## 2. Face Recognition with Color 2-Dimensional Principal Component Analysis-Convolutional Neural Network (C2D-CNN) Model

### 2.1. Overview of the Proposed Method

**Figure 1:**
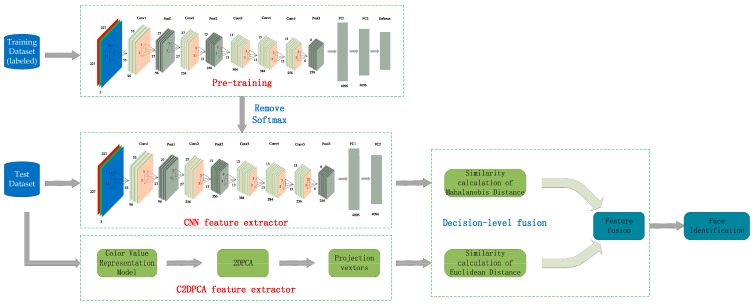
The flow diagram for face recognition using color 2-dimensional principal component analysis-convolutional neural network (C2D-CNN).

The face recognition method using a C2D-CNN model under decision-level fusion is divided into three parts: feature extraction for CNN, feature extraction for color 2DPCA, and decision-level fusion. The algorithm flow is shown in Figure 1.

Figure 1 shows the overall flow of the proposed method, C2D-CNN. In the proposed algorithm, firstly, the input image is scale normalized. Then, our algorithm is divided into three parts, including CNN feature extraction, color 2DPCA feature extraction and decision-level fusion. Furthermore, CNN and color 2DPCA are respectively applied to extract features from the normalized images. Among them, the CNN model is trained on the FRGC (Face Recognition Grand Challenge) v2.0 dataset to obtain the depth features. Color 2DPCA extracts handcrafted features that contain richer color information and spatial information. In order to fuse the feature vectors, Mahalanobis distance and Euclidean distance are respectively used to calculate the similarity of the feature vectors. Finally, the similarity weight ratio is set (the setting method will be described in detail in the Section 3.3.3) to fuse the feature vectors in decision level.

### 2.2. Feature Extraction with CNN

**Figure 2:**
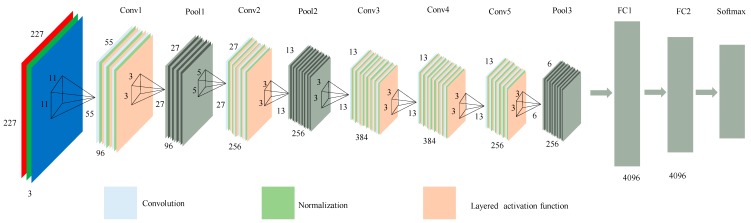
The structure of the CNN network based on the face recognition.

The CNN model proposed in this paper consists of convolution layer, normalization layer, layered activation function layer, probabilistic max-pooling layer, and fully connected layer. In this algorithm, CNN is first pre-trained with large samples, and then the pre-trained CNN model (Softmax classifier is removed) is used as feature extractor to extract facial features. The structure of the CNN network is shown in Figure 2.

The input of the convolutional neural network are color face images which have been scale normalized. Firstly, the input image is convoluted through several different convolution kernels to manipulate and extract the image features. The filter coefficients are obtained automatically during the training process and are dependent on the characteristics of the input training images. Then, the convolutional operation results are normalized in the normalization layer, which effectively prevents the network from over-fitting and ensures effective gradient descent. In order to increase the non-linear expression ability of the network, the normalized data is input into the layered activation function layer. Finally, the result of the activation function is pooled, retaining the most significant features and improving the model’s distortion tolerance. After several convolutional layers and pooled layers, the acquired salient features are processed through the fully connected layer to obtain CNN feature representation results.

The pre-training of the CNN mainly includes the process of forward learning network parameters and back propagation of network parameters.

#### 2.2.1. Forward Propagation of CNN Network

The parameters of the network were learned through the forward propagation. In each layer, the input features are calculated as follows:

(1) $$k^{\left(l+1\right)}=LA(BL(F^{\left(l+1\right)}k^{\left(l\right)}+b^{\left(l+1\right)}))$$

*k*^(*l*)^ is the vector output of the *l*-th layer, *k*^(*l*+1)^ is the vector output of the (*l* + 1)-th layer, *F*^(*l*+1)^ is a matrix of the inter-layer linear coefficients, *b*^(*l*+1)^ is the vector composed of biases, *LA* (·) is the layered activation function, and *BL* (·) is the normalization algorithm proposed in this paper.

(1) Normalization algorithm

In the training process of the CNN, due to the changes of network parameters, the distribution of input data in each layer might change, which in turn leads to different learning rates required for different dimensions. While pre-training the network, it is usually necessary to select the minimum learning rate, thus preventing the network from over-fitting and ensuring the effective decline of the gradient. To address this problem, we use normalization to process the data of each dimension and make it satisfy the same distribution. Normalization can prevent the occurrence of over-fitting when still using a large learning rate.

Supposing that the output of a layer contained *η* dimensions, *x* = {*x*^(1)^, *x*^(2)^,…, *x*^(*η*)^}, the normalization was applied independently to each dimension. Then, we take any dimension as an example for explaining. In a mini-batch sample with the size of *s*, the data of the mini-batch can be defined as: *B_x_* = *x*_1_, *x*_2_, …, *x*_s_. The normalized sample data is *B_y_*, *B_y_* = *y*_1_, *y*_2_, …, *y*_s_, *y_i_* (*i* ∈ [1, *s*]) obeys the standard normal distribution with zero mean and unit variance.

In Algorithm 1, the initial value of *μ_B_* was set as 0 and the initial value of ${\sigma_{B}}^{2}$ was set as 1. *γ* is the learning rate related to the momentum values *φ* and *ψ*, having been set as 0.01 by default. The specific combination coefficient *φ* and *ψ* were learned from the data, thus *φ* and *ψ* are self-adaptive to the input data. During the test phase, $\mu_{B}$ and ${\sigma_{B}}^{2}$ took on the final training value.

**Algorithm 1** NormalizationInput: CNN Network and mini-batch *B_x_*
Output: Normalized sample data *B_y_*1. Mini-batch mean: $\mu =\frac{1}{s}\sum_{i=1}^{s}x_{i}$2. Mini-batch variance: $\sigma^{2}=\frac{1}{s}{\sum_{i=1}^{s}\left(x_{i}-\mu \right)}^{2}$3. Normalized value: $y_{i}=\frac{x_{i}-\mu }{\sigma }$4. Update the global mean: $\mu_{B}=(1-\varphi )*\mu_{B}+\varphi *\mu$5. Update the global variance: $\sigma_{B}{}^{2}=(1-\psi )*\sigma_{B}{}^{2}+\psi *\sigma^{2}$6. Update the momentum value *φ*: $\varphi =\varphi -\gamma \frac{\partial L}{\partial \varphi }$7. Update the momentum value *ψ*: $\psi =\psi -\gamma \frac{\partial L}{\partial \psi }$

During the training phase, the back-propagation gradient of the normalization layer was determined by the chain rule:

(2) $$\frac{\partial L}{\partial \sigma^{2}}=-\frac{1}{2}\sum_{i=1}^{s}\frac{\partial L}{\partial y_{i}}(x_{i}-\mu ){\left(\sigma^{2}\right)}^{\frac{3}{2}}$$

(3) $$\frac{\partial L}{\partial \mu }=(\sum_{i=1}^{s}\frac{\partial L}{\partial y_{i}}\frac{-1}{\sigma })+\frac{\partial L}{\partial \sigma^{2}}\frac{-2\sum_{i=1}^{s}\left(x_{i}-\mu \right)}{s}$$

(4) $$\frac{\partial L}{\partial x_{i}}=\frac{\partial L}{\partial y_{i}}\frac{1}{\sigma }+\frac{\partial L}{\partial \sigma^{2}}\frac{2(x_{i}-\mu )}{s}+\frac{1}{s}\frac{\partial L}{\partial \mu }$$

Given the output loss function *L*, we can learn the combination coefficient *φ* and *ψ*. The back propagation for this learning is given by:

(5) $$\frac{\partial L}{\partial \varphi }=\frac{\partial L}{\partial \mu_{B}}\frac{\partial \mu_{B}}{\partial \varphi }=\frac{\partial L}{\partial \mu_{B}}(\mu -\mu_{B})$$

(6) $$\frac{\partial L}{\partial \psi }=\frac{\partial L}{\partial (\sigma_{B}{}^{2})}\frac{\partial (\sigma_{B}{}^{2})}{\partial \psi }=\frac{\partial L}{\partial (\sigma_{B}{}^{2})}(\sigma^{2}-\sigma_{B}{}^{2})$$

Due to the parallelism of the calculation, the normalization in the mini-batch was more effective than in all dimensions, so the data was normalized by using mini-batch to satisfy the same distribution. In addition, ideally, the mean and variance should be specific to all dimensions, which was impractical. Therefore, the mean and variance of all dimensions was estimated using the mean and variance of the mini-batch data, with it all and partially linked by updating the global variance and mean, thus simplifying the normalized calculation process. In the experiment, it was found that the recovery operation in BN had little effect on improving the speed of the network training. To further improve the convergence rate, the normalization layer removed the recovery operation, which worked better than BN.

The normalization on the mini-batch data alleviated the problem of Internal Covariate Shift and non-linear saturation, which greatly reduced the training time of the network and effectively accelerated the convergence of the network.

(2) Layered activation function

**Figure 3:**
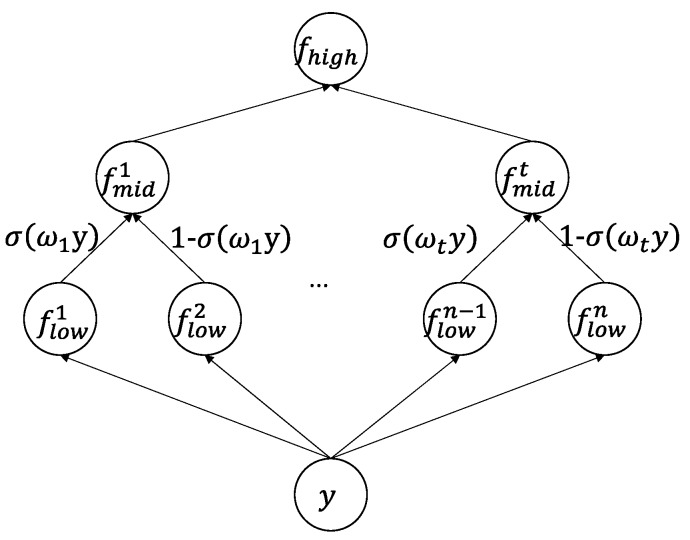
The structure of the layered activation function.

The purpose of the activation operation is to introduce the non-linearity into the CNN network. Furthermore, we introduce the layered activation function, which might address 3 problems: (1) adaptivity is introduced to the activation function; (2) improve the non-linear expression ability; (3) solve the problems such as gradient saturation, gradient diffusion. The structure of the layered activation function is shown in Figure 3.

Figure 3 shows a three-level structure in which low-level nodes are associated with learnable basic activation functions. Each middle-level node corresponds to a combined output of a pair of low-level nodes in a binary tree structure, and a high-level node corresponds to the overall output. Among them, *y* is the normalized input parameter, and *f_low_*, *f_mid_*, *f_high_* denote low-level nodes, middle-level nodes, and high-level node functions, respectively. The three-level node function is given by:

(7) $$f(y)=\left\{ \begin{array}{ll} f_{low}^{n}(y), & low-nodes\\ \sigma (\omega y)f_{mid}^{k,left}(y)+(1-\sigma (\omega y))f_{mid}^{k,right}(y), & middle-nodes\\ \underset{1\leq k\leq t}{\max}f_{mid}^{k}(y), & high-nodes \end{array}\right.$$

where, $f_{low}^{n}$(**·**) is represents the *n*-th low-level node function (*f_prelu_*(·) or *f_elu_*(·)), σ(**·**) is sigmoid function, $f_{mid}^{k,left}$(**·**) and $f_{mid}^{k,right}$(**·**) are the left or right child node functions of the *k*-th middle node respectively. The basic activation function is given by:

(8) $$f_{prelu}(y)=\left\{ \begin{array}{ll} y, & y>0\\ \alpha y, & y\leq 0 \end{array}\right.$$

(9) $$f_{{}_{elu}}(y)=\left\{ \begin{array}{ll} y, & y>0\\ \beta (e^{y}-1), & y\leq 0 \end{array}\right.$$

In the formula, *α* and *β* are the weights of the negative part of control, which are learnt from data. The initial value of *α* is set as 0.25 and *β* is set as 1.0 [46]. $f_{mid}^{k}$(**·**) represents the *k*-th middle-node function, given by:

(10) $$f_{mid}(y)=\sigma (\omega y)f_{prelu}(y)+(1-\sigma (\omega y))f_{elu}(y)$$

The high-level node function *f_high_*(·) is the maximum of *t* middle-nodes function.

**Figure 4:**
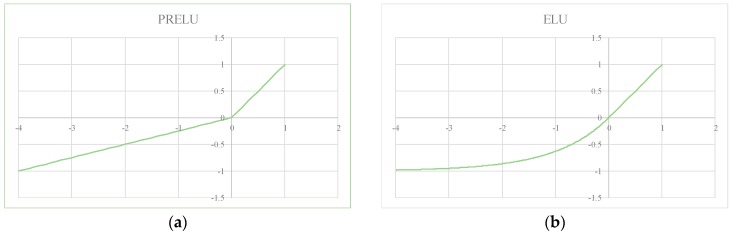
Activation functions of low-nodes: (**a**) PRELU; (**b**) ELU.

The advantages of the two basic activation functions are: The *f_prelu_*(·) allows the slope of the negative part to be learned from the data, which improves the expression ability of the activation function; the linear component of *f_elu_*(·) makes it possible to mitigate the gradient disappearance and the soft-saturation can reduce the slope from a predefined or fixed threshold to zero, which can accelerate model learning. The basic activation function is shown in Figure 4.

The middle-level node function *f_mid_*(·) can adaptively choose the combination coefficient of the two basic activation functions. Specifically, we instead learn a gating mask ω, rather than directly learning a combination coefficient. The output of the gating mask and *y* is jointly fed to a sigmoid function to generate the combination coefficient.

The output of the layered activation function $f^{\mathrm{high}}\;=\;{}_{1\leq k\leq t}^{\max}f_{mid}^{k}$ (y) is the maximum of *t* middle-level nodes. The competition mechanism is introduced to enhance the non-linearity of the activation operation. Only the activation neuron with the highest activation can be activated while other neurons were inhibited.

The algorithm learns the layered activation function in a data-driven manner. *f_prelu_*(·) and *f_elu_*(·) functions are introduced to solve the gradient diffusion problem. By adaptively combining them, the layered activation function has a good adaptability. The competition mechanism is introduced to improve the ability to learn non-linear transformation.

(3) Probabilistic max-pooling

Next is the pooling layer, whose main task is to blur the features so as to obtain the invariance of shift and scale. The state of neurons in this layer is determined by the upper local “accepted domain” and the pooling rules of the upper layer. In general, the minimum, mean, median and maximum of the neuron response values in the “accepted domain” are taken as the pooling rules. Although these rules can achieve the above requirements, however, they are irreversible. In the process of back propagation, the loss of feature representations is inevitable, which directly restricts the robustness of CNN. A probabilistic max-pooling approach [47] is applied so that the feature information is preserved to maximum extent while maintaining feature invariance: when there is, as long as a neuron is opened, the response will be generated by the sample value *S*; otherwise, *S* does not respond, which can be expressed as:

(11) $$P(S=1|k)=\sum_{i,j\in C}\exp({\left(F^{l}*k\right)}_{ij}+b_{l})/(1+\sum_{i,j\in C}\exp({\left(F^{l}*k\right)}_{ij}+b_{l}))$$

#### 2.2.2. Back Propagation (BP) of CNN Networks

To improve the self-adaptability of the new network, the back propragation (BP) algorithm [48] was used to adjust the parameters in reverse. In view of the fact that the parameters of the network update very slowly by using the variance loss function, the cross-entropy cost function [49] was adopted, whose advantage lies in that large errors can lead to quick updates of the network’s parameters while small errors can slowly update the network’s parameters. As for the voiceprint training sample set with the size of *N*, *e* = {(*e*^(1)^, *v*^(1)^), …, (*e*^(*N*)^, *v*^(*N*)^)}, the cross entropy cost function was defined as:

(12) $$\Gamma (\theta )=-\frac{1}{N}\sum_{i=1}^{N}\left[\nu^{\left(i\right)}\log(o^{\left(i\right)})+(1-\nu^{\left(i\right)})\log(1-o^{\left(i\right)})\right]$$

where *o*^(*i*)^ denotes the actual output corresponding to the input *e*^(*i*)^, and *v*^(*i*)^ denotes the category tag corresponding to the *i*-th group of data, *v*^(*i*)^ ∈ {1, 2, 3, …, *k*}, *k* is the number of face categories, and the back-propagation gradient of the convolutional parameters *w* and *b* were determined by the following formulas:

(13) $$\frac{\partial }{\partial w^{\left(i\right)}}\Gamma (\theta )=\frac{1}{N}\sum_{i=1}^{N}x^{\left(i\right)}(o^{\left(i\right)}-v^{\left(i\right)})$$

(14) $$\frac{\partial }{\partial b^{\left(i\right)}}\Gamma (\theta )=\frac{1}{N}\sum_{i=1}^{N}\left(o^{\left(i\right)}-v^{\left(i\right)}\right)$$

The calculation formulas of the updated parameters *w*^(*i*)^ and *b*^(*i*)^ are as follows:

(15) $$w_{i}^{l}=w_{i}^{l}-\rho \frac{\partial }{\partial w_{i}^{l}}E^{\tau }$$

(16) $$b_{i}^{l}=b_{i}^{l}-\rho \frac{\partial }{\partial b_{i}^{l}}E^{\tau }$$

where, *ρ* is the learning rate, and *E^τ^* is the error of training face samples for the current batch face images.

#### 2.2.3. Feature Extraction

**Figure 5:**
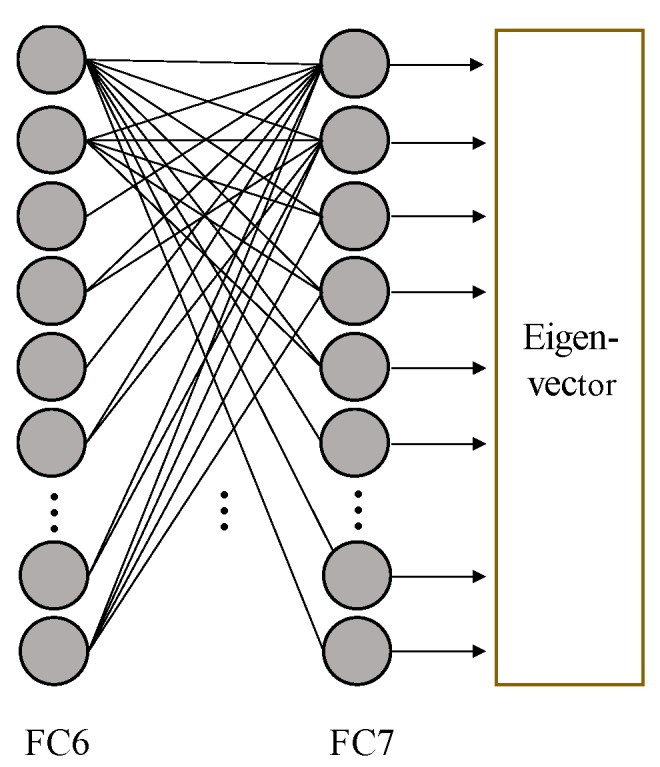
The structure of the fully connected layer.

In this algorithm, a pre-trained CNN model is used as a feature extractor to extract the facial features. The 4096-dimensional output of the last fully connected layer is used as the eigenvector extracted by CNN. The structure of the fully connected layer is shown in Figure 5.

The output of the fully connected layer is determined by the following formula:

(17) $$\rho^{\left(l+1\right)}=\psi (W^{\left(l+1\right)}\rho^{\left(l\right)}+b^{\left(l+1\right)})$$

*ρ*^(*l*)^ is the output vector of the *l*-th layer, *ρ*^(*l*+1)^ is the output vector of the (*l* + 1)-th layer, *W*^(*l*+1)^ is a matrix of the inter-layer linear coefficients, *b*^(*l*+1)^ is the vector composed of biases, and *ψ*(·) is the activation function.

### 2.3. Feature Extraction with Color 2-Dimensional Principal Component Analysis (2DPCA)

Traditional 2DPCA [50] and PCA [51] need to convert the original image to a grayscale version before extracting the feature, so the color information is usually ignored. Color 2DPCA introduces the concept of color value (taking R, G, B color components as a whole) and applies the existing 2DPCA framework to color face recognition directly [52]. Compared with most of the previous methods which make use of three color channels respectively, color 2DPCA can obtain more color information and spatial information.

First, the composite vector representation of the color face image *A* is defined as:

(18) $$A={\left(c_{1},c_{2},\ldots ,c_{n}\right)}^{T}$$

Among them, the basic element *c_i_* is denoted as the *i*-th color value (the color vector of the pixel). Once the basic operations are defined for color values, the color values can be regarded as normal scalar values for computing, now we define the basic operations of the color value: (1) addition: the sum of the color values is the sum of the two color vectors; (2) subtraction: subtraction of the color value is the difference between the two color vectors; (3) multiplication: the product of the two color values are the inner product of the two color vectors. In this algorithm, multiplication is mainly used to calculate the covariance of two features, reflecting the correlation of two color values. If the result of multiplication is zero, they are independent, otherwise they are positive or negative correlation.

Then, the composite vector *A* of the image is projected onto the vector *X*, the projection feature vector *Y* of *A* can be obtained as follows:

(19) Y=AX

Suppose each person has *M* samples, with $\bar{A}$ representing the average value of *M* composite vectors, the sample matrix is defined as:

(20) $$D={\left[{\left(A_{1}-\bar{A}\right)}^{T},{\left(A_{2}-\bar{A}\right)}^{T},\ldots ,{\left(A_{M}-\bar{A}\right)}^{T}\right]}^{T}$$

where *D* is a composite matrix (the basic elements of *D* are color value rather than scalar values). Each element in row represent a centered sample and *T* represents the transpose. According to PCA, the optimal projection vector can be obtained by calculating *D^T^D*.

(21) $$D^{T}D=\sum_{j}^{M}{\left(A_{j}-\bar{A}\right)}^{T}(A_{j}-A)=M\times G_{t}$$

Among them, the sample covariance matrix *G_t_* is defined as:

(21) $$G_{t}=\frac{1}{M}\sum_{j=1}^{M}{\left(A_{j}-\bar{A}\right)}^{T}(A_{j}-\bar{A})$$

In this way, 2DPCA can be applied to find the optimal projection of the image composite vector *A*. The total scatter *J*(*X*) of the projection sample is introduced to detect the discriminative ability of the projection vector. *J*(*X*) can be represented by the trace of the covariance matrix of the projection vector *Y*:

(23) $$\begin{array}{cc} J(X) & =tr(S_{X})\\ & =X^{T}E\left\{{\left(A-E(A)\right)}^{T}(A-E(A))\right\}X\\ & =X^{T}G_{t}X \end{array}$$

The optimal projection axis *X_opt_* = {*X_1_*, *X_2_*, …, *X_d_*} is the former *d* orthogonal column vectors.

(24) $$\left\{ \begin{array}{l} \left\{X_{1},X_{2},\ldots ,X_{d}\}=\arg\max J(X\right)\\ X_{i}^{T}X_{j}=0,i\neq j,i,j=1,2,\ldots ,d \end{array}\right.$$

Finally, the projection feature matrix of the image is obtained by using the best projection axis. The projection matrix of image *A* is:

(25) $$Y^{i}=AX_{opt}=[AX_{1},AX_{2},\ldots ,AX_{d}]=[Y_{1}^{i},Y_{2}^{i},\ldots ,Y_{d}^{i}]$$

*Y_i_* is the projection feature matrix extracted from the color 2DPCA.

### 2.4. Decision-Level Fusion

Because it is difficult to obtain good recognition performance when there is a big difference between test and training set, inspired by complementary theory, we combine color 2DPCA features with CNN features for face recognition. Color 2DPCA can extract rich color and spatial information. It has good robustness even when there is a big difference between test and training set. The Mahalanobis is widely used in face recognition for CNN [53,54], and the Euclidean distance is popular for PCA, 2DPCA and related algorithm [55,56]. They are respectively applied to the calculation of similarity. Finally, fusion of similarity weight is performed at the decision level.

For the CNN feature, suppose $w_{1}^{\prime \prime }$ and $w_{2}^{\prime \prime }$ are two learned eigenvectors for the gallery and query face images, respectively. The similarity *G^CNN^* of the CNN eigenvector is calculated by the following formula:

(26) $$G^{CNN}=\frac{{\left(w_{1}^{\prime \prime }\right)}^{T}\cdot w_{2}^{\prime \prime }}{\left|w_{1}^{\prime \prime }|\times |w_{2}^{\prime \prime }\right|}$$

where, |·| is the L2 norm, *T* is the transpose.

For the similarity of color 2DPCA features, the similarity between gallery image *A_i_* and query image *A_j_* is calculated as formula (27).

(27) $$d(A_{i},A_{j})={\sum_{k=1}^{d}\left\|Y_{k}^{i}-Y_{k}^{j}\right\|}^{2}$$

where, $\Sigma_{k=1}^{d}$||$Y_{k}^{i}$ *−* $Y_{k}^{j}$|| is represents the Euclidean distance between two eigenvectors. Decision-level convergence is determined by the following equation:

(28) $$G=\Omega G^{\;CNN}+(1-\Omega )G^{\;C2D}$$

Among them, *G* is the fusion similarity, *G^C^*^2*D*^ is the normalized similarity of color 2DPCA, and Ω is the weight of similarity (Ω < 1).

## 3. Experimental Results and Analysis

In this section, the experimental environment and the details of our paper will be described. All of the ideas of comparative experiments come from the proposed algorithm of this article, and they are applied to evaluate our method and demonstrate the correctness of our algorithm. The experiments are performed on the LFW (Labeled Faces in the Wild) and FRGC face databases and the results will be compared with the recognition rates in the relevant literature.

### 3.1. Dataset

**Figure 6:**
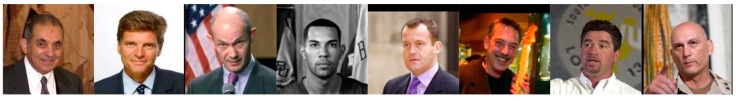
The software interface based on face recognition.

In this paper, the LFW face database [57] and FRGC v2.0 face database [58] were selected for experiments to evaluate the face recognition algorithm proposed in this paper. The LFW database contains 13,233 face images collected from the Internet, among them, there are 1680 persons have two or more images and 4096 people have only one image. LFW is mainly used for research on face recognition under unrestricted conditions and has become the benchmark for academic evaluation of recognition performance. It can fully reflect the changes of face in real conditions, such as illumination, gesture, expression, background, gender etc. Figure 6 shows some of the pictures in the LFW database.

**Figure 7:**
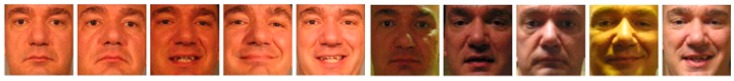
The software interface based on face recognition.

We report the averaged result over 10 runs of View2. The FRGC v2.0 face database includes 12,776 training images (including 6388 uncontrolled images and 6388 controlled images), 16,028 controlled object images and 8014 uncontrolled query images. The controlled images are of good quality, but the uncontrolled images are poor, such as illumination changes, low resolution and blurring, etc. FRGC Experiment 4 has been reported to be the most challenging FRGC experiment, so it is chosen to assess the face verification rate at the false accept rate of 0.1%. Figure 7 shows some of the pictures in the FRGC database.

In this paper, the recognition rate will be applied to evaluate the performance of the algorithm. *N_r_* is the number of faces matched correctly, and *N_a_* is the total number of face samples (including the number matching correct and mismatch faces). *R* is recognition rate, giving:

(29) $$R=\frac{R_{r}}{R_{a}}$$

### 3.2. Experiment Setting

#### 3.2.1. Training Details

**Figure 8:**
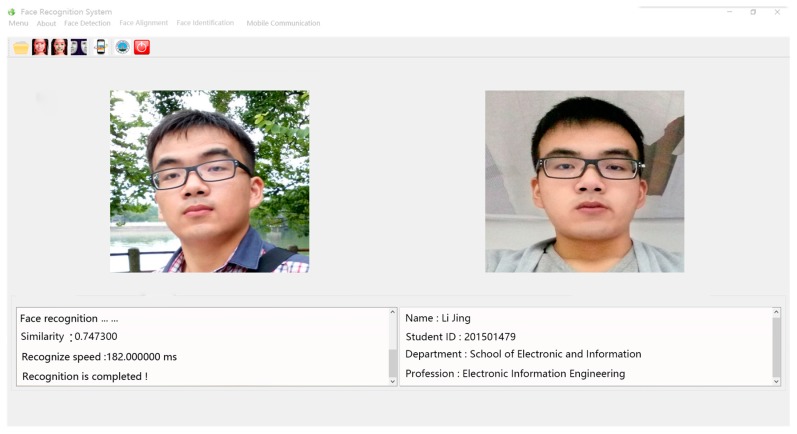
The software interface based on face recognition.

The experiments were carried out in the operating system of ubuntu1604, with a NVIDIA GEFORCE GTX 1050 GPU, memory size of 16 GB, CPU as I7-7700, software platform on python 3.5, and tensorflow 1.2.1, the interface software for a cross-platform Qt machine. As is shown in Figure 8, software for face recognition was developed by using the proposed algorithm.

In this part, we will introduce the training and experiment details. In the following experiments, we will compare three models: CNN-0 (AlexNet), CNN-1 (AlexNet + BN), CNN-2 (Our model). In addition, we also report the accuracy under the LFW training and identification protocol. GTX 1050 GPU is applied to carry on parallel acceleration calculations. Other details about the pre-training network are shown in Table 1.

In the following part, we will introduce the setting of the color 2DPCA. For the color 2DPCA, we need to determine its dimensions and the number of training and test samples, according to the actual experiment, when the dimension is greater than 30, its accuracy has tended to be stable and not increasing. We will select the training samples from two benchmark databases as the training set, and we know that the higher the proportion of training samples, the higher the accuracy. Therefore, we set the proportion of 1:3. The details about decision-level fusion are shown in the Table 2.

For the proposed algorithm C2D-CNN, there are a few other parameters that will be obtained in Section 3.3.

In the following experiment, when the similarity is greater than or equal to 0.7, the proposed algorithm considers face recognition to be successful. The following experimental data are the average result.

#### 3.2.2. Data Augmentation Details

To train these three network models, 12776 FRGC database training images are selected as the training set. But the training set is too small to train a network model. To magnify the number of training samples, for each training sample of size m × m we extract all possible crops of size (m − 4) × (m − 4). Each crop is also flipped along its vertical axis yielding a total of 2 × 5 × 5 = 50 kinds of crops. Then the crops are re-sized back to same size.

#### 3.2.3. Fine-Tuning Details

Generally, there is a big difference between the dataset of the target task and the pre-trained dataset, regardless of the number of categories or image pattern. In the retrieval task of the target dataset, it is often difficult to achieve better performance by using the pre-trained CNN model to extract the visual features of the image. Therefore, in order to make the pre-trained CNN model parameters more suitable for the feature extraction of the target dataset, we use the image of the target dataset to fine-tune the pre-trained CNN model parameters. Fine-tuning training refers to retraining the CNN model on the FRGC training set. In the training process, only the output layer weights are initialized using Gaussian random numbers, and other weights of the CNN model are initialized using the corresponding weights of the pre-training model.

The difference from the pre-training is that a small learning rate should be set in the first 7 layers of the network, which can ensure that the parameters obtained through the pre-training CNN model are not destroyed during the fine-tuning process. For the last layer, setting a higher learning rate ensures that the entire network quickly converges on the target dataset to the new optimal point. For the CNN-0 network, in order to ensure the normal convergence of the network, the lr (learning rate) is reduced to 1/10 for every 20 epochs.

In Section 3.4.5, we will compare the effectiveness of fine-tuning and decision-level fusion. More details about the pre-training network are shown in the Table 1. The details about Fine-Tuning are shown in the Table 3.

### 3.3. Acquisition of Testing Parameter

**Figure 9:**
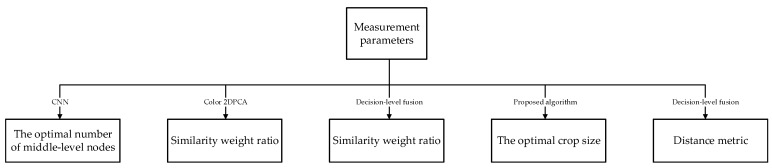
Acquisition of testing parameters.

In this part, to give the proposed algorithm better robustness and recognition performance, firstly, we need to dtermine certain parameters of our proposed algorithm, and then we can safely apply it to specific recognition tasks. The parameters to be obtained are as follows: the number of layered activation function middle-nodes, the optimal feature dimension, fusion weight ratio, and image size. As the similarity weight ratio and feature dimension may vary with the benchmark face database used, two databases are used for experiments. For experiments unrelated to the face database, only the FRGC database will be applied. The specific flow is shown in Figure 9.

#### 3.3.1. Acquisition of the Optimal Number of Middle-Nodes in Layered Activation Function

**Figure 10:**
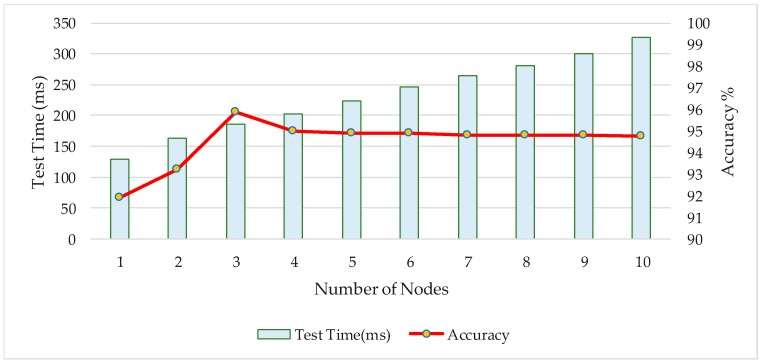
Experiment of layered activation function middle-nodes setting.

In this experiment, we evaluated the influence of the number of the middle-level nodes on the recognition performance for the CNN network and expected to obtain the optimal number of nodes. The benchmark database FRGC was selected as the test dataset. Ten groups of experiments were carried out, the number of middle-level nodes is respectively: 1, 2, 3, 4, 5, 6, 7, 8, 9, 10. We evaluated the recognition rate and the averaged test time. The experimental results are shown in Figure 10.

As is shown in Figure 10, when the number of middle-level nodes is three, the optimal recognition rate is achieved. As the number of nodes increases, the recognition rate tends to be stable in a small area, however, the test time is significantly increased. In all the next experiments, the number of middle-level nodes will be set to three.

#### 3.3.2. Acquisition of the Optimal Feature Dimension of Color 2DPCA in Different Benchmark Database

**Figure 11:**
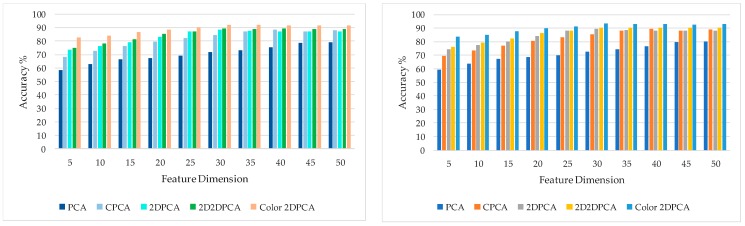
Evaluation results under various feature dimension in two benchmark databases: (**a**) FRGC; (**b**) LFW.

In this experiment, we compared the performance of the PCA-related algorithm under various feature dimensions and expected to obtain the optimal feature dimension of color 2DPCA. The benchmark database FRGC and LFW were selected as the test dataset. Ten groups of experiments were carried on. We compared 5 kinds of PCA related algorithm: PCA, CPCA (color PCA), 2DPCA (Two-Dimensional PCA), 2D2DPCA (2 Dimensional 2 Directional PCA), color 2DPCA. Among them, PCA is a basic dimension reduction algorithm, furthermore, CPCA can extract color feature, 2DPCA can extract spatial information and significantly reduce the dimensionality of features. The improvement of 2D2DPCA is that the transformation in ordinary 2DPCA only extracts the features in the rows of the data matrix, but the 2D2DPCA adds the transformation to the columns. The experimental results are shown in Figure 11.

As can be seen from the experimental results shown in Figure 11, compared with other PCA dimensionality reduction algorithms, the recognition performance of the color 2DPCA algorithm is dominant in the two benchmark databases. Coincidentally, under different databases, the PCA and CPCA algorithms have the highest recognition accuracy when the dimension is 45, but the 2DPCA correlated algorithm tends to have the highest recognition accuracy when the dimension is 30, which can achieve the great recognition result at lower dimensions compared with the PCA-related algorithm. That is not surprising, color 2DPCA can effectively extract the color feature and spatial feature. Hence, in all the next experiments, the feature dimension will be set to 30.

#### 3.3.3. Acquisition of the Optimal Similarity Weight Ratio in Different Benchmark Database

**Figure 12:**
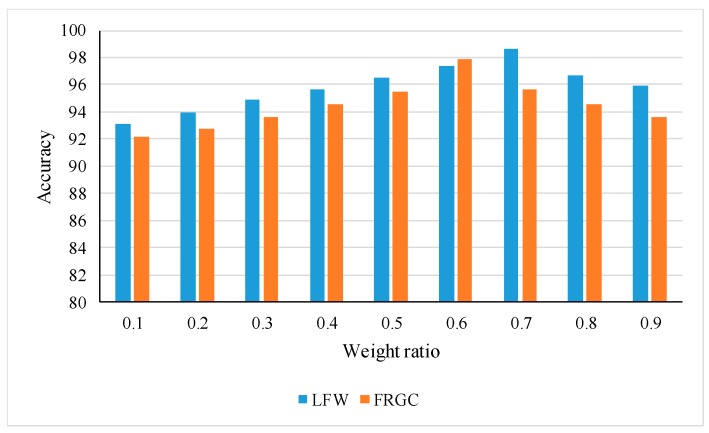
The face recognition rate of different similarity weights.

In this experiment, it was known that the similarity *G* was determined by Formula (28), and the optimal similarity weight Ω under different datasets was evaluated, respectively. FRGC face database and LFW database are selected as query image sets, and the color 2DPCA is trained and tested according to the proportion of 1.5:1. The size of face images is resized to 227 × 227. There are nine groups of comparison experiments, respectively. The value of Ω values were: 0.1, 0.2, 0.3, 0.4, 0.5, 0.6, 0.7, 0.8, 0.9. The experimental results are shown in Figure 12.

From the experimental results shown in Figure 12, for the LFW database, the optimal weight is Ω = 0.7 and the recognition rate is 98.64%. For the FRGC face database, the optimal weight is Ω = 0.6 and the optimal recognition rate is 97.86%. For different face database, the weight ratio is changing. When the weight ratio is maximum or minimum, i.e., decision-level fusion is at a disadvantage, the recognition rate is low. We can safely draw a conclusion that decision-level convergence is a great help to improve the recognition performance. Color 2DPCA can effectively extract color and spatial information from color face images, and still has good robustness for the test and training sets under the conditions of large differences, which can make up for the defect of poor recognition performance of the CNN network.

#### 3.3.4. Acquisition of the Optimal Crop Size for Different CNN Models

In this experiment, the image size is related to both the recognition performance and computational cost of CNN network, to evaluate the performance of the CNN under different crop size, FRGC face database was selected as test dataset. There were five groups experiments: 127 × 127, 160 × 160, 192 × 192, 227 × 227, 250 × 250. Due to the random data argumentation, the smaller the image size, the bigger the randomness in training. We compared the recognition performance of different image size under the FRGC protocol. The experimental results are shown in Table 4. 

As can be seen from the experimental results shown in Table 4, the optimal recognition performance was obtained when the pixel size is 227 × 227. 

#### 3.3.5. Acquisition of the Optimal Distance Metric Method

**Figure 13:**
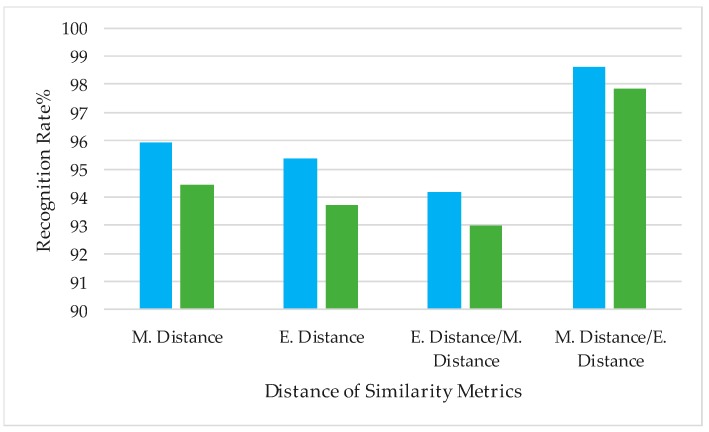
The accuracy under the different combinations of various distance metrics.

In this experiment, we evaluated the influence of the distance metrics method for the proposed C2D-CNN method and expected to obtain the optimal combination of the distance metrics. The benchmark database LFW, FRGC were selected as the test datasets. Four groups of experiments were carried out, the combinations were respectively: Mahal. Distance used only, Euclid. Distance used only, Euclid. Distance for CNN and Mahal. Distance for color 2DPCA, Mahal. Distance for CNN and Euclid. Distance for color 2DPCA. Since the distance metric algorithm has changed, the optimal similarity weight ratio has changed. According to the experiment, we obtained the optimal similarity weight ratio of the four distance algorithm combinations under two benchmark databases, 0.7/0.8 0.5/0.6, 0.5/0.6, 0.6/0.7, respectively. To embody the difference of the accuracy for various combinations more carefully, the experiment was performed under the condition of the best weight ratio. The results are shown in Figure 13.

According to the experimental results shown in Figure 13, the combination of Euclidean distance for CNN features and Mahalanobis distance for color 2DPCA features can achieve optimal recognition performance. Different distance metric methods will lead to different recognition accuracy. Compared to Euclidean distance, Mahalanobis distance has a better performance for CNN features, and Euclidean distance is the most commonly used similarity metric method for 2DPCA and its related algorithms.

### 3.4. Testing Result and Discussion

**Figure 14:**
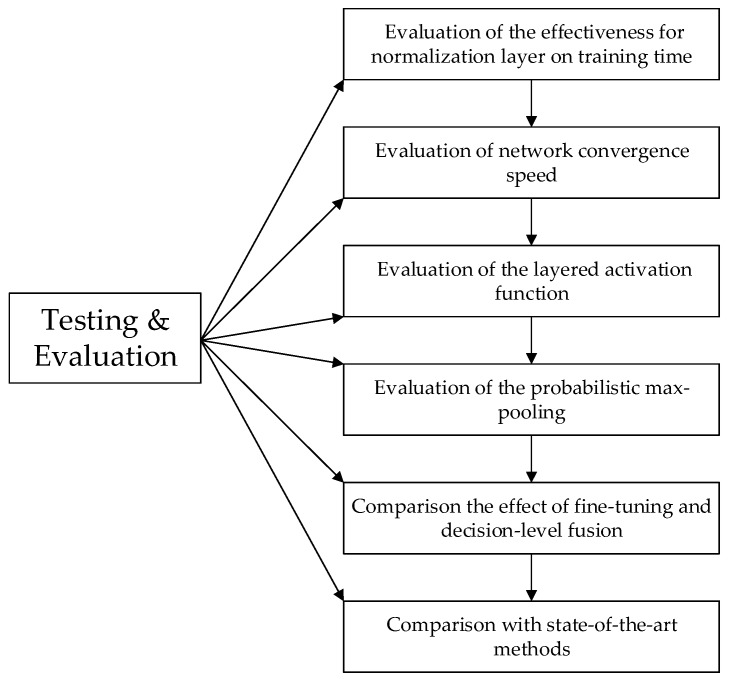
Flow chart of experiment.

In this part, the proposed C2D-CNN algorithm will be compared with other state-of-the-art algorithms to verify the effectiveness and robustness of our algorithm. Firstly, we will evaluate the validity of the proposed and introduced normalization layer, layered activation function, and probabilistic max-pooling, respectively. Secondly, we will return to the original intention of the proposed novel idea, the comparison of the fine-tuning and decision-level fusion. Finally, we will compare the proposed algorithm with the state-of-the-art method. The specific flow is shown in Figure 14.

#### 3.4.1. Evaluation of the Performance of Normalization Layer on Training Time

**Figure 15:**
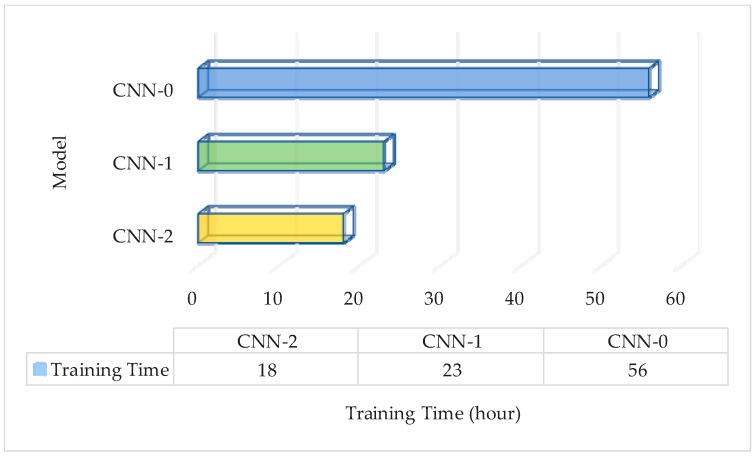
Comparison of the pre-training time of the three models.

In this experiment, we evaluated the effectiveness of normalization on training time. The overall network cost penalty was set as 0.01. There were three groups of experiments: CNN-0, CNN-1 and CNN-2. The learning rate of CNN-1 and CNN-2 was set as 0.05, and the learning rate of CNN-0 was set as 0.01. We set the value of weight ratio to be 0.6 on the FRGC database. The network was trained according to the experimental setup. The experimental results are shown in Figure 15.

As can be seen from the experimental results shown in Figure 15, the normalization layer demonstrated a good performance in the process of network training. The training time was reduced by 67.9% compared to the unnormalized CNN-0 network, and a 21.7% decrease compared to the CNN-1 network for batch normalization. This is because the normalization layer can normalize data to standard normal distribution, which can effectively prevent overfitting, and can choose a larger learning rate to accelerate network convergence. Compared with batch normalization, the normalization has significant advantages. (1) The normalization layer removed the recovery operation, thus reducing the consumption of GPU memory and computation cost; (2) we used the mean and variance of the mini-batch data to estimate the mean and variance of all dimensions, linking them by updating the global variance and mean; (3) when the global variance and mean value were updated, the specific combination coefficient *φ* and *ψ* were learned from the data, and thus *φ* and *ψ* were self-adaptive to the input data.

#### 3.4.2. Evaluation of Network Convergence Speed

**Figure 16:**
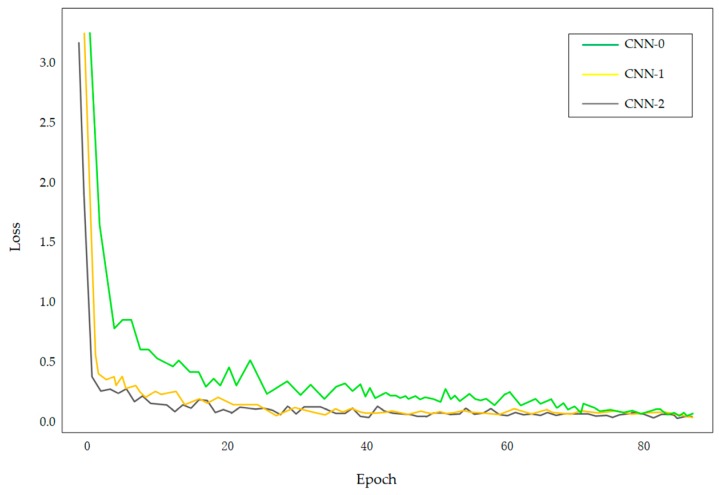
Comparison of the convergence speed of the two networks.

In this experiment, three groups of experiments were carried out to evaluate the effects of the normalization layer on the convergence rate of the model. Compared with CNN-0, normalization and batch normalization were introduced to the CNN-1 and CNN-2 models, respectively. The CNN models with and without normalized process were pre-trained with the FRGC face databases, respectively. The network was trained according to the experimental setup. We set the value of weight ratio to be 0.6 on the FRGC database. According to the loss value, the convergence rate of the model can be evaluated. The experimental results are shown in Figure 16.

According to the experimental results shown in Figure 16, with the epoch, both of three models gradually converged, however, the convergence speed of the CNN-0 network without normalization is significantly slower than the CNN-1 and CNN-2 networks which were processed by normalization or batch normalization. Compared with CNN-1 network, although the two curves are close, the convergence speed of CNN-2 network still has obvious advantages. And as the iterative process proceeded, the loss value of the network tended to be stable. The experimental results verified that the normalization layer can effectively accelerate the convergence of the network.

#### 3.4.3. Evaluation of the Performance of Layered Activation Function

**Figure 17:**
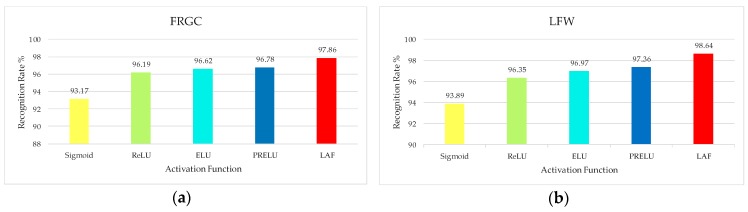
Comparison of activation functions: (**a**) FRGC face database; (**b**) LFW face database.

To evaluate the activation performance of the layered activation function, we did experiments on two benchmark datasets. We selected the FRGC uncontrolled image and LFW database as the test set. The number of middle-level nodes in the layered activation function layer was set to three. We set the value of weight ratio to be 0.6 on FRGC database and 0.7 on the LFW database. Five groups of experiments were performed: Sigmoid, ReLU, ELU, PRELU and LAF (layered activation function). We compared the effectiveness of five activation functions. Different activation functions will directly affect the recognition performance of the CNN model. In this experiment, we compared the recognition rate of the proposed C2D-CNN algorithm under different activation functions. The experimental results are shown in Figure 17.

As is shown in Figure 17, identification performance of the network with layered activation function is better than the other basic activation functions. The success of the layered activation function is due to its adoption of a hierarchical acquisition strategy. The strategy of hierarchical activation, which introduces learning and adaptation into the activation operation to make it adapt to the normalized data, can give full play to the basic activation function and the role of the hierarchy. Firstly, by adaptively combining the basic activation function, we can solve the existing gradient problem well and get better activation effect. Then, the competition mechanism is introduced to enhance the non-linearity of the activation operation.

#### 3.4.4. Evaluation of the Performance of the Probabilistic Max-Pooling

**Figure 18:**
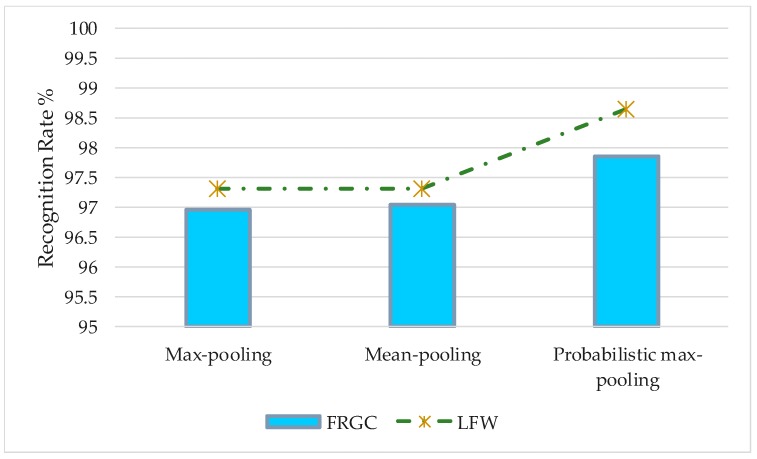
Comparison of pooling method.

To evaluate the effectiveness of the pooling method on robustness and recognition performance, two benchmark databases, FRGC and LFW, were chosen for test set. In this experiment, we compared the accuracy of the proposed algorithm C2D-CNN under different pooling functions. We set the value of weight ratio to be 0.6 on FRGC database and 0.7 on the LFW database. The experiment includes three groups of comparison experiments: max-pooling, mean-pooling, and probabilistic max-pooling. The experimental results are shown in Figure 18.

As is shown on Figure 18, the performance of the network with probabilistic max-pooling is better than other pooling method and reached 97.86%. The mean and maximum of the neuron response values in the “accepted domain” are usually taken as the pooling rules. However, in the process of back propagation, the loss of feature representations is inevitable, which directly restricts the robustness of the CNN. A probabilistic max-pooling approach is applied so that the feature information is preserved to the maximum extent while maintaining feature invariance. Compared with the other pooling method, probabilistic max-pooling can achieve a better pooling performance.

#### 3.4.5. Comparison of the Effectiveness of Fine-Tuning and Decision-Level Fusion

**Figure 19:**
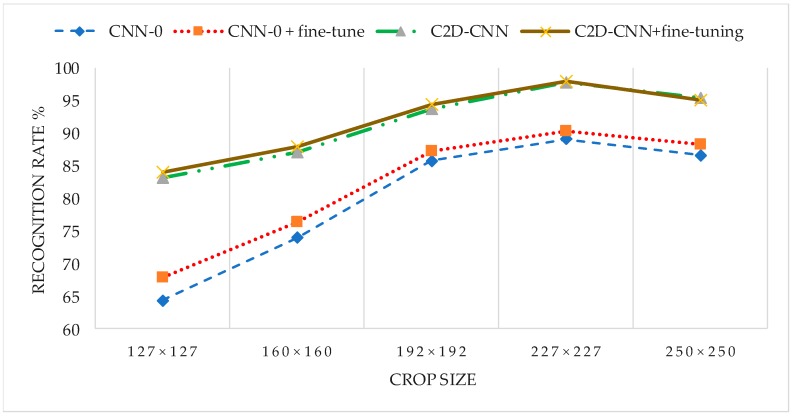
Evaluation the effect of fine-tuning and decision-level fusion on improving the performance of CNN.

In this experiment, we evaluated the recognition performance of the C2D-CNN model and compared the effectiveness of decision-level fusion and fine-tuning in improving recognition accuracy. As there is no label information to use for fine-tuning the pre-trained model, we only test the performance on the FRGC database, and chose to experiment with FRGC Experiment 4. This included four groups of experiments: CNN-0, CNN-0 + fine-tuning, C2D-CNN, C2D-CNN + fine tuning; The similarity weight is set to 0.6. The experimental results are shown in Figure 19.

As can be seen from the experimental results shown in Figure 19, the difference of recognition performance between the fine-tuned network and original network is very small, especially for the proposed model in this article, which is almost no difference. The experimental results show that when there is big difference between test and training set, fine tuning cannot solve the problem of low recognition rate well, but the introduction of the color 2DPCA feature is more effective than trimming CNN in improving the performance of pre-training the CNN model. This is because the C2D-CNN model based on decision-level fusion combines two features that have complementary properties which integrates the advantages of the color 2DPCA feature and CNN feature so as to make the fused similarity more accurate and generalization ability enhanced.

#### 3.4.6. Comparison with State-of-the-Art Methods

We evaluated our method in the close-set and open-set face identification tasks, following the protocol in [58]. The close-set identification protocol reports the Rank-1 identification accuracy and the open-set identification reports the DIR (Detection and Identification Rate) at FAR (Detection and Identification Rate) equal to 1%. The comparisons with the state-of-the-art method are shown in Table 5. The proposed C2D-CNN model achieves state-of-the-art performance in face identification tasks.

The performance results of the proposed method clearly demonstrated that C2D-CNN is very effective for the improvement of face recognition; what’s more, the CNN model we proposed and applied has achieved optimal results in the process of identification and training. All of the architectures performed very well on two benchmark datasets. The diversity of these datasets demonstrates that our model is effective for a wide range of activity recognition tasks. The C2D-CNN method using decision-level fusion has a better recognition performance than the fine-tuning network when there are big differences between test set and training set in viewpoint, expression, illumination, or other factors.

There are two main reasons for the superb performance of the proposed method C2D-CNN. First, in the design of CNN network architecture, we fully consider the network training and identification process, and constantly optimize the architecture of CNN, proposed and introduced the normalization layer, layered activation function layer, and the probabilistic max-pooling, cross-entropy cost function, which has a good performance in improving the accuracy of face recognition and training speed. Second, before we came up with C2D-CNN, we considered that it was not enough to optimize the performance of recognition only from the improvement of network architecture. However, we could fundamentally solve the problems that CNN cannot avoid by fully considering the complementary information of different features. We consider two complementary features, which are fused at the decision level, and the experimental results show that this fusion is successful.

## 4. Conclusions and Future Work

In this paper, we proposed a novel robust face recognition using a deep C2D-CNN model under decision-level fusion to solve face recognition when there is a big difference between the test and the training set. Inspired by the idea of complementarity, two features are fused at the decision level. In addition, to improve the performance of CNN network, we proposed a novel network structure. Firstly, we introduced a normalization layer in CNN to speed up the network convergence and shorten the training time. Secondly, the layered activation function was introduced to make the activation function adaptive to the normalized data. Finally, probabilistic max-pooling was applied to preserve the feature representations in order to maximize extent while maintaining feature invariance. In the future, we will focus on the deeper integration of deep learning and traditional machine learning approaches to address face recognition problems in more severe environments.

## Figures and Tables

**Table 1:** The details of the CNN model.

	CNN-0	CNN-1	CNN-2
Learning rates	0.01	0.05	0.05
Dropout	0.5	---	---
Weight decay	0.001	0.001	0.001
Momentum	0.9	0.9	0.9
No. of epochs	80	25	20
Batch _size	512	512	512
Pooling method	Max-pooling	Max-pooling	Probabilistic max-pooling
Activation function	ReLU	ReLU	LAF
other	---	Batch Normalization	Normalization

**Table 2:** The details of decision-level fusion.

	CNN-2	Color 2DPCA
Input	Color face images	Color face images
Output dimensions	4096	30
Matching strategy	Mahalanobis distance	Euclidean distance
Weight ratio	Depending on the sample database	

**Table 3:** The details of Fine-Tuning.

	CNN-0	CNN-2
Learning rates	Conv1-FC2: 0.005 Softmax: 0.02	Conv1-FC2: 0.02 Softmax: 0.05
Dropout	0.5	---
Weight decay	0.001	0.001
Momentum	0.9	0.9
No. of epochs	60	20
Batch _size	512	512
Pooling method	Max-pooling	Probabilistic max-pooling
Activation function	ReLU	LAF
other	---	Normalization

**Table 4:** The performance of the CNN with different crop sizes.

Crop Size	Accuracy %			
	CNN-0	CNN-0 + Fine-Tune	CNN-2	CNN-2 + Fine-Tune
127 × 127	64.32	67.96	83.24	84.01
160 × 160	73.94	76.31	87.14	87.97
192 × 192	85.75	87.19	93.77	94.37
227 × 227	89.12	90.23	97.86	98.02
250 × 250	86.63	88.27	95.34	95.15

**Table 5:** The performance of our method and state-of-the-art methods on LFW under the identification protocol.

Method	Rank-1 (%)	DIR@1% FAR (%)
COST-S1	56.7	25.0
COTS-S1 + S2	66.5	35.0
DeepFace	64.9	44.50
WSTFusion	82.5	61.90
Our Method	91.98	63.34
